# Identification and Functional Analysis of the Nocardithiocin Gene Cluster in *Nocardia pseudobrasiliensis*

**DOI:** 10.1371/journal.pone.0143264

**Published:** 2015-11-20

**Authors:** Kanae Sakai, Hisayuki Komaki, Tohru Gonoi

**Affiliations:** 1 Medical Mycology Research Center, Chiba University, Chiba, Japan; 2 Biological Resource Center, National Institute of Technology and Evaluation, Chiba, Japan; University of San Agustin, PHILIPPINES

## Abstract

Nocardithiocin is a thiopeptide compound isolated from the opportunistic pathogen *Nocardia pseudobrasiliensis*. It shows a strong activity against acid-fast bacteria and is also active against rifampicin-resistant *Mycobacterium tuberculosis*. Here, we report the identification of the nocardithiocin gene cluster in *N*. *pseudobrasiliensis* IFM 0761 based on conserved thiopeptide biosynthesis gene sequence and the whole genome sequence. The predicted gene cluster was confirmed by gene disruption and complementation. As expected, strains containing the disrupted gene did not produce nocardithiocin while gene complementation restored nocardithiocin production in these strains. The predicted cluster was further analyzed using RNA-seq which showed that the nocardithiocin gene cluster contains 12 genes within a 15.2-kb region. This finding will promote the improvement of nocardithiocin productivity and its derivatives production.

## Introduction

Actinomycetes are Gram-positive soil bacteria that produce various secondary metabolites. For example, metabolite production is well documented in *Streptomyces* species and many studies on secondary metabolite production have been conducted using *Streptomyces* strains [1, 2]. *Nocardia* species, which are mostly opportunistic human and animal pathogens, are also members of the actinomycetes bacteria. Although secondary metabolite production in *Nocardia* species is less well studied, a recent genome-based analysis revealed that the number of secondary metabolite gene clusters in *Nocardia* species is comparable with that of *Streptomyces* species [3]. This suggests that *Nocardia* species may also be a sources of secondary metabolites.

**Fig 1:**
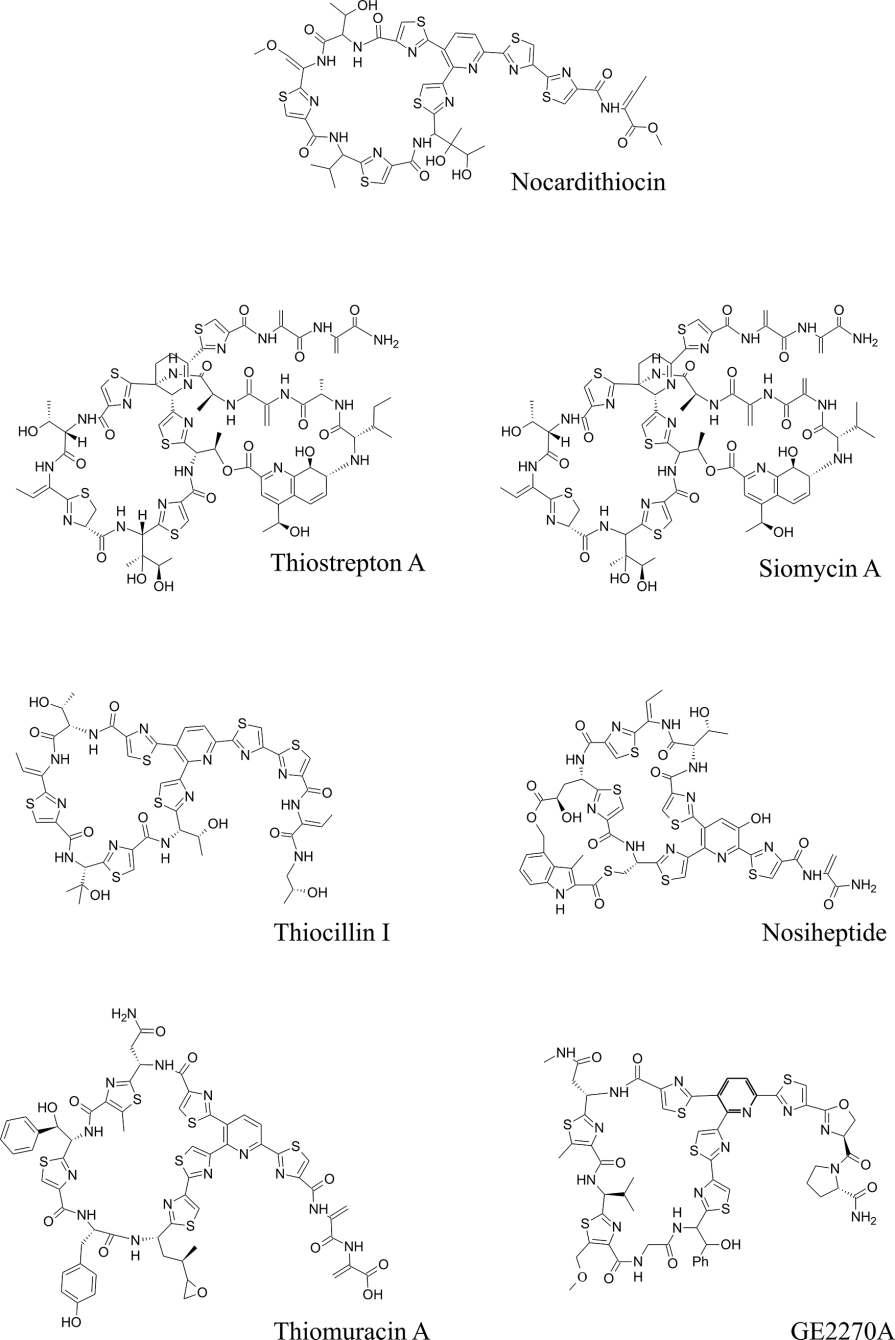
Structure of nocardithiocin and other thiopeptides.

Recently, Mukai et al. identified a thiopeptide compound, nocardithiocin, from *Nocardia pseudobrasiliensis* [4] (Fig 1).Thiopeptides (thiazolyl peptides) are highly modified sulfur-rich peptides synthesized by the ribosome. These compounds all contain a central nitrogen-containing six-membered ring, which serves as the scaffold for at least one macrocycle and a tail. These compounds possess a wide range of bioactivities, including antimicrobial, anticancer, and antiplasmodial effects. In addition to these clinically promising activities, this family of compounds was recently highlighted, because many members of thiopeptides show potent activity against drug-resistant pathogens, including methicillin-resistant *Staphylococcus aureus*, penicillin-resistant *Streptococcus pneumoniae*, and vancomycin-resistant enterococci [5–9]. Nocardithiocin also has relatively high antibiotic activity against acid-fast bacteria, and effectively suppresses growth of rifampicin-resistant *Mycobacterium tuberculosis* [4].

Peptide-based natural products are synthesized by ribosome pathways or nonribosomal peptide synthase pathways. Thiopeptides biosynthesis begins with ribosomally-synthesized precursor peptides and follows several modifications, such as cyclization and dehydration, to form the macrocycle structure. Further side-chain modifications produce the final complex products [10, 11]. Several thiopeptide biosynthetic gene clusters have already been identified using the sequences of putative precursor peptides, cyclodehydratases, and modification enzymes as markers [12–14]. Other than the gene coding for precursor peptide, all thiopeptide gene clusters reported to date contain several core sets of genes that participate in the heterocyclization and dehydration of core peptides [11].

Here, we identified the gene cluster responsible for nocardithiocin biosynthesis in whole-genome sequence of *N*. *pseudobrasiliensis*. The relationship between nocardithiocin production and the predicted cluster was confirmed using gene disruption and complementation analyses.

## Materials and Methods

### Strains and media

All *N*. *pseudobrasiliensis* strains were obtained from the Medical Mycology Research Center, Chiba University, Japan (Table 1). *Escherichia coli* DH5α was used for vector construction and propagation. *N*. *pseudobrasiliensis* strains were cultivated at 30°C or 37°C in Brain-Heart Infusion (BHI: Becton Dickinson and Company, Tokyo, Japan) broth or 2% agar plates supplemented with 1% glucose and 1% glycerol (BHIgg). For nocardithiocin production, Czapek-Dox (CD) medium consisting of 0.6% NaNO_3_, 0.052% KCl, 0.152% KH_2_PO_4_, 1% glucose, 2 mM MgSO_4,_ and trace elements, was used.

**Table 1:** Bacterial strains used in this study.

IFM No.	Origin	Country	Nocardithiocin production*
**0623**	No record (N. R.)	Japan	+
**0624**	N. R.	USA	+
**0756**	N. R.	USA	-
**0757**	N. R.	USA	+
**0758**	N. R.	USA	+
**0759**	N. R.	USA	+
**0760**	N. R.	USA	+
**0761**	N. R.	USA	+
**0896**	sputum	Japan	+
**10098**	sputum	Thailand	±
**10099**	pus	Thailand	±
**10700**	pus	Japan	-
**11475**	blood	Japan	+
**11477**	bronchoalveolar lavage	Japan	±

*The degree of nocardithiocin production was roughly divided into three levels: +, obvious production; ±, very slight production; -, no production.

### Nocardithiocin production and detection

The strains were pre-cultured in 5 mL of BHIgg medium at 37°C for 72 h. The pre-cultures were then used to inoculate (1% of medium, v/v) 10 mL of CD medium and cultivated at 30°C for 7 days. Following cultivation, the culture supernatants were extracted with equal volumes of ethyl acetate, and the ethyl acetate layer was evaporated in vacuo. Amounts of nocardithiocin in the samples were analyzed by HPLC using a Wakopack Wakosil-II 3C18 HG column (Wako Pure Chemical Industries, Osaka, Japan). The column was developed using a gradient of 10–100% acetonitrile in water over 50 min at a flow rate of 1 mL/min and monitored by a fluorescence detector (excitation at 350 nm and emission at 450 nm) (RF-20A/RF-20Axs, Shimadzu Corp., Kyoto, Japan).

### Whole genome sequencing of *N*. *pseudobrasiliensis* IFM 0761

Genomic DNA was extracted from 48–72 h cultures grown in BHIgg broth. The collected cells were treated with 1 mg/mL lysozyme in DNA extraction buffer (200 mM Tris-HCl, 25 mM NaCl, 25 mM EDTA, pH8.5) at 37°C for 30 min. The lysed cells were further treated by 1% SDS at 70°C for 10 min. Following the cell lysis, genomic DNA was extracted using a phenol-chloroform extraction method. A 4-μg of genomic DNA was then treated using a Nextera DNA Sample Prep kit (Illumina, Tokyo, Japan) according to the manufacturer’s instructions. The quality of the libraries was confirmed by Agilent 2100 Bioanalyzer using a High Sensitivity DNA kit (Agilent Technologies, Santa Clara, CA, USA), while a Quant-iT PicoGreen dsDNA Reagent kit (Invitrogen, Carksbad, CA, USA) was used to quantify the libraries. The denatured library mixture was sequenced using a Miseq sequencer (Illumina) according to the manufacturer’s instructions, using a Miseq Reagent Kit v2 (300 cycle; Illumina). The raw sequence data were assembled using the CLC Genomics Workbench (CLC Bio, Tokyo, Japan) and annotated by the Microbial Genome Annotation Pipeline (MiGAP; http://www.migap.org). The genome sequence was deposited in the DDBJ Sequence Read Archive (DRA) under the accession numbers BBXO01000001−BBXO01002032.

### Prediction of the nocardithiocin biosynthetic gene cluster from genome sequence

An essential gene for thiopeptide biosynthesis, cyclodehydratase, was amplified by PCR using the genomic DNA as a template and degenerate primer set (Thio-F (5′-TACGAGACCTCCAAYGGNTGYGCN-3′) and Thio-R (5′-GTGGCCRAASGTCATNGG-3′), final concentration was 1 pmol/μL) [10]. PCR amplification was conducted as follow, 94°C for 2 min, followed by 35 cycles of 94°C for 30 sec, 52°C for 30 sec and 68°C for 30 sec using Quick Taq HS DyeMix (TOYOBO, Osaka, Japan). The amplified fragment was sequenced and its homologous region was found in the whole genome sequence of *N*. *pseudobrasiliensis* IFM 0761, and the flanking region of its homologous sequence was investigated to find other genes which may participate in nocardithiocin biosynthesis.

### Construction of vectors for disruption and complementation of *notL*

The disruption vector for the *notL* (the putative cyclodehydratase) was constructed as follows: upstream and downstream fragments of *notL* were amplified using PCR using the notL-upF/-upR and notL-downF/-downR primer sets, respectively, and *N*. *pseudobrasiliensis* IFM 0761 DNA as the template. The kanamycin resistance gene *aph* was amplified using the Laph-F/-R primer set with pNV18.1 as the template. KOD-Plus-Neo (TOYOBO) was used as the enzyme for all PCRs. The three fragments were then ligated into the *Hin*cII site of pUC19 using GeneArt Seamless Cloning and Assembly Enzyme Mix (Life Technologies, Tokyo, Japan) according to the manufacturer’s instructions.

For the *notL* complementation vector, *notL* was expressed under the control of the *ermE* promoter (P*ermE*) and *rrnB* terminator (T*rrnB*). The P*ermE* fragment was amplified using the primer set PermE-F/-R and pKU470 as the template. The *notL* fragment was amplified using the primer set notL-compF/-R and *N*. *pseudobrasiliensis* IFM 0761 DNA as the template. The T*rrnB* fragment was amplified from pRU1701 (Addgene, Cambridge, MA, USA) using the TrrnB-F/-R primer set. All the three PCR fragments were ligated and cloned into the *Hin*cII site of pNV38.1 (containing a chloramphenicol resistance gene) using GeneArt Seamless Cloning and Assembly Enzyme Mix. The disruption and complementation vectors were confirmed by sequencing. All primer sequences are listed in Table 2.

**Table 2:** Primers used in vector construction.

Primer name	Sequence 5′ – 3′
notL-upF	TTGTCGGCTTCGTCGGTGTGTATGGGTAGC
notL-upR	GACTCTAGAGGATCCGGATCCTCTAGAGTCACCGCGAACACGATCGGTAC
notL-downF	GCATGCCTGCAGGTCAGGGTGGTGTAGGTGGTGAG
notL-downR	GACCAAGCGACGCGACCGGGCTACCGGTAG
Laph-F	GTAGCCCGGTCGCGTCGCTTGGTCGGTCAT
Lapa-R	CATACACACCGACGAAGCCGACAATCCACC
PermE-F	TGCCTGCAGGTCGCGAGTGTCCGTTCGAGT
PermE-R	CGAGCGCCTCATCGCTGGATCCTACCAACC
notL-compF	GCGAGTGTCCGTATGAGGCGCTCGGTAGTC
notL-compR	CTTTCGTTTTATTCAGGGGAACGGATGCGG
TrrnB-F	CCGTTCCCCTGAATAAAACGAAAGGCTCAG
TrrnB-R	TCCTCTAGAGTCATAAAACGAAAGGCCCAG

### Transformation of N. pseudobrasiliensis

*N*. *pseudobrasiliensis* was cultivated in 10 mL BHIgg medium at 37°C overnight. Cells were collected by centrifugation and rinsed with ice-cold water, followed by 10% glycerol, and suspended in ice-cold 10% glycerol. A 50-μL aliquot of cell suspension was then transferred to a chilled electroporation cuvette (2 mm gap width) and mixed with 1-μL of vector suspension. After pulsing at 12.5 kV/cm in a MicroPulser electroporator (Bio-Rad Laboratories, Hercules, CA, USA), the cuvette was placed on ice for 2 min, and then 900 μL of BHIgg was added to the cuvette. After incubating at 37°C for 3 h, cells were plated onto BHIgg plates containing 100 μg/mL of neomycin or chloramphenicol, and further incubated at 37°C for 2−3 days.

Resulting single cross-over strains were confirmed by colony PCR, and candidate colonies were cultivated in BHIgg medium without neomycin at 37°C for 2− 3 days to obtain the second cross-over strains. The cultivated cells were diluted and plated onto BHIgg plates without neomycin. The resulting colonies were picked and duplicate-plated onto BHIgg plates with and without neomycin. The neomycin-sensitive putative double cross-over colonies were confirmed by PCR and Southern blot analysis.

### Transcriptional analysis

RNA was extracted from cells cultured under both nocardithiocin-producing and non-producing conditions. The RPMI 1640 medium (TaKaRa, Shiga, Japan) supplemented with 10% fetal bovine serum (FBS; Gibco Inc., Tokyo, Japan) was used for the nocardithiocin-producing conditions, and RPMI 1640 medium without FBS was used for the non-producing conditions. Cells pre-cultivated cells on BHIgg medium at 37°C for 72 h were inoculated into the nocardithiocin-producing and non-producing media and then incubated at 37°C under a 5% CO_2_ atmosphere for 7 days. Following incubation, the cells were collected and suspended in ISOGEN RNA extraction solution (Nippon Gene Co., Tokyo, Japan). Resuspended cells were homogenized using a MagNA Lyser (Roche Diagnostics, Tokyo, Japan) at a speed of 7,000 for 20 s, and RNA was extracted according to the ISOGEN protocol. Following DNase treatment, 2 μg of total RNA were treated with a Ribo-Zero Magnetic Kit (Epicentre, Madison, WI, USA) to remove ribosomal RNA. For the construction of the RNA-seq library, obtained mRNA samples were treated with a SureSelect Strand Specific RNA library preparation kit (Agilent Technologies) according to the manufacturer’s protocol. Constructed libraries were sequenced with HiSeq system (Illumina) using TruSeq Rapid SR Cluster Kit-HS (Illumina). The resulting data were mapped against sequenced genome data of *N*. *pseudobrasiliensis* IFM0761 using the CLC Genomics Workbench.

## Results

### Selection of a nocardithiocin high-producing strain

**Fig 2:**
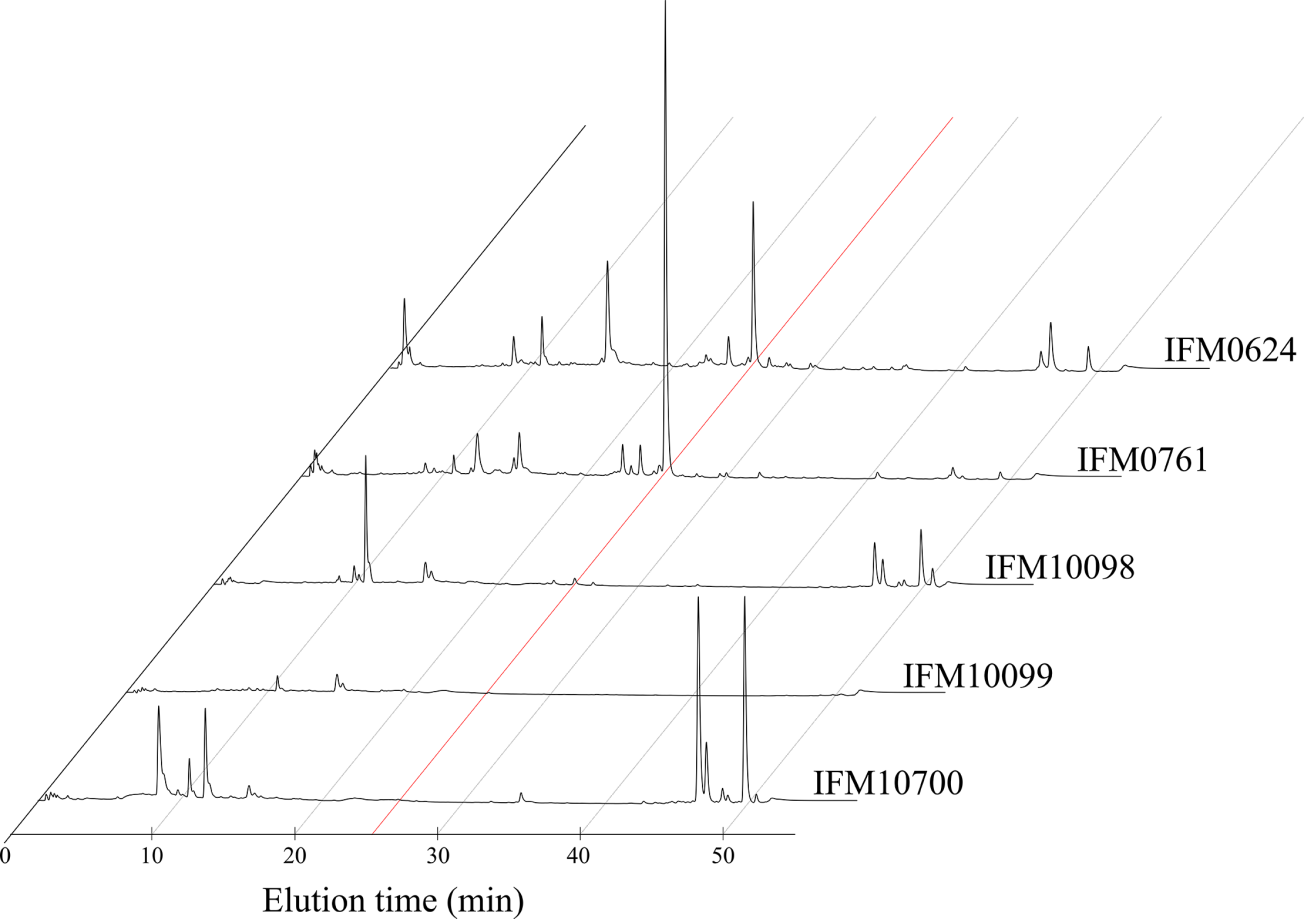
Confirmation of nocardithiocin production. HPLC analysis of the nocardithiocin production in representative *Nocardia pseudobrasiliensis* IFM strains. In total, 14 strains were tested (Table 1). The peak pattern did not differ considerably between the strains, therefore representative data are shown. The red line indicates the elution time of nocardithiocin.

Prior to attempting to identify the nocardithiocin biosynthetic gene cluster, we confirmed the nocardithiocin production of 14 *N*. *pseudobrasiliensis* strains stocked at the Medical Mycology Research Center, Chiba University, Japan (Table 1). Under our experimental conditions, two strains did not produce nocardithiocin, while *N*. *pseudobrasiliensis* IFM 0761 produced the greatest amount of nocardithiocin, as estimated by HPLC fluorescent peaks (Fig 2). The nocardithiocin peak was confirmed by its UV and MS spectrum (S1 Fig). Because higher production levels detection of the compound easier, IFM 0761 was used in further experiments.

### Identification of the nocardithiocin gene cluster

**Fig 3:**
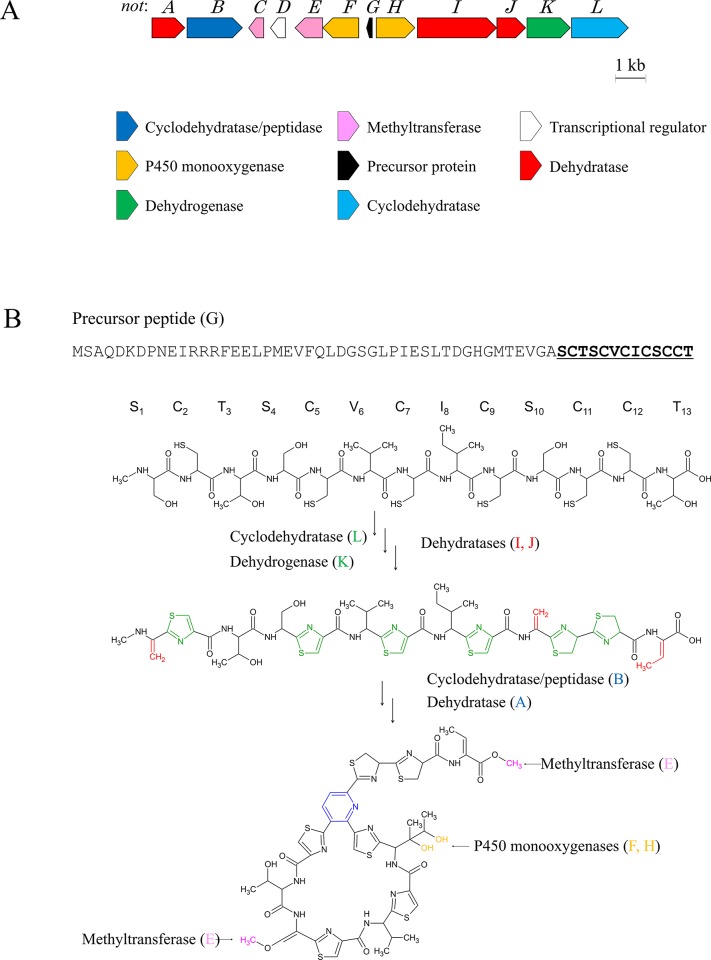
Nocardithiocin gene cluster and predicted biosynthesis scheme. (A) Organization of genes within the cluster. Each gene is color-coded by its putative function. (B) Proposed nocardithiocin biosynthesis pathway based on the chemical structure and putative gene functions. The dehydrogenation reaction forms a thiazole ring (green), dehydration produces dehydroamino acids (red), and the cyclization of the precursor peptide at serines S1 and S10 forms a pyridine ring. Further modifications by P450 monooxygenase (yellow) and methyltransferase (pink) are also indicated.

To locate the nocardithiocin biosynthetic gene cluster in the *N*. *pseudobrasiliensis* genome, we first tried to identify the cyclodehydratase genes, which are relatively conserved among bacterial strains and required for thiopeptide biosynthesis [10]. Using degenerate primer sets, amplified a putative cyclodehydratase gene fragment and then sequenced it. A search of the NCBI databases using the BLASTx algorithm confirmed that the 674-bp amplified fragment showed high homology to the cyclodehydratase gene of *Nonomuraea* species Bp3714-39 (TpdG: 55% identity) [15]. Based on this result, we obtained the sequences of the amplified region from the *N*. *pseudobrasiliensis* IFM 0761 genome, and tentatively predicted that a cluster of 12 genes (*notA*−*notL*) within a15.2-kb region was responsible for nocardithiocin biosynthesis (Fig 3A). This cluster contained one precursor peptide, one transcriptional regulator, and other modification enzymes needed for heterocyclization and side chain modification. Although gene organization within the cluster differed from other reported thiopeptide gene clusters, the genetic composition was similar (S2A Fig). Furthermore, the amino acid sequence of the precursor peptide of the predicted gene cluster correlated with the nocardithiocin structure. Based on the predicted functions of the 12 genes, a biosynthetic scheme was proposed (Fig 3B).

### Gene disruption and nocardithiocin production

**Fig 4:**
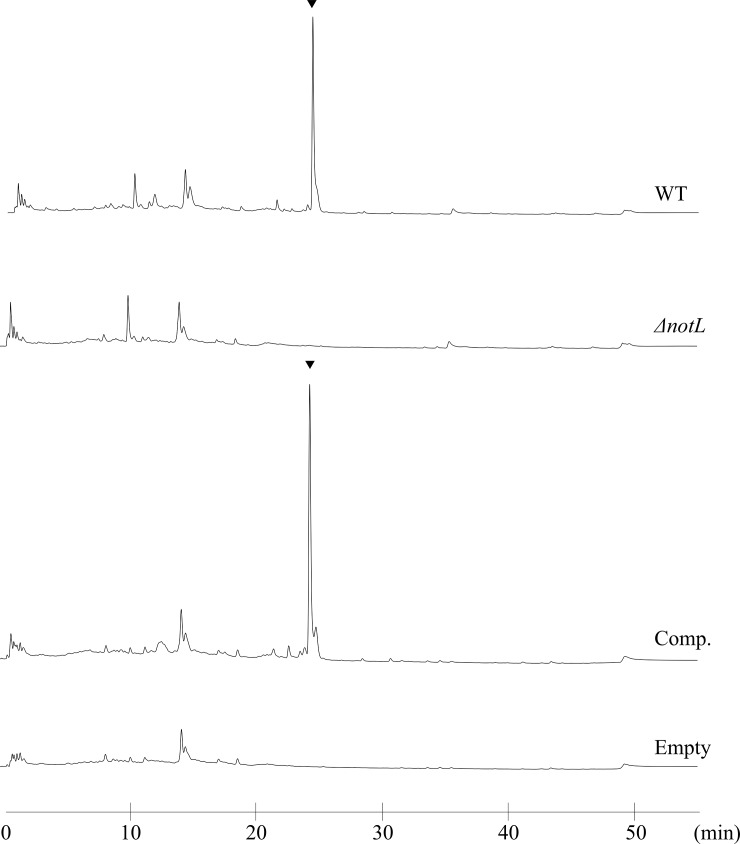
Nocardithiocin production. Nocardithiocin production in the wild-type (WT), *notL* disruptant (*ΔnotL*), and complement strain (Comp.) was analyzed by HPLC.*ΔnotL* strain containing an empty vector (Empty) was used as the control. The arrowheads indicate the nocardithiocin peak.

To confirm the relationship between nocardithiocin production and the predicted gene cluster, the putative cyclodehydratase gene *notL* was disrupted by replacement with an antibiotic resistance gene. The genotypes of the resulting disruptants were confirmed by Southern blotting and sequencing (data not shown). Nocardithiocin production in the disruptants was analyzed by HPLC (Fig 4), which revealed complete loss of nocardithiocin production. Additionally, nocardithiocin production was restored in the complementation strains (Fig 4, lower panel), suggesting that the predicted gene cluster was responsible for nocardithiocin production.

### Transcriptional analysis

**Fig 5:**
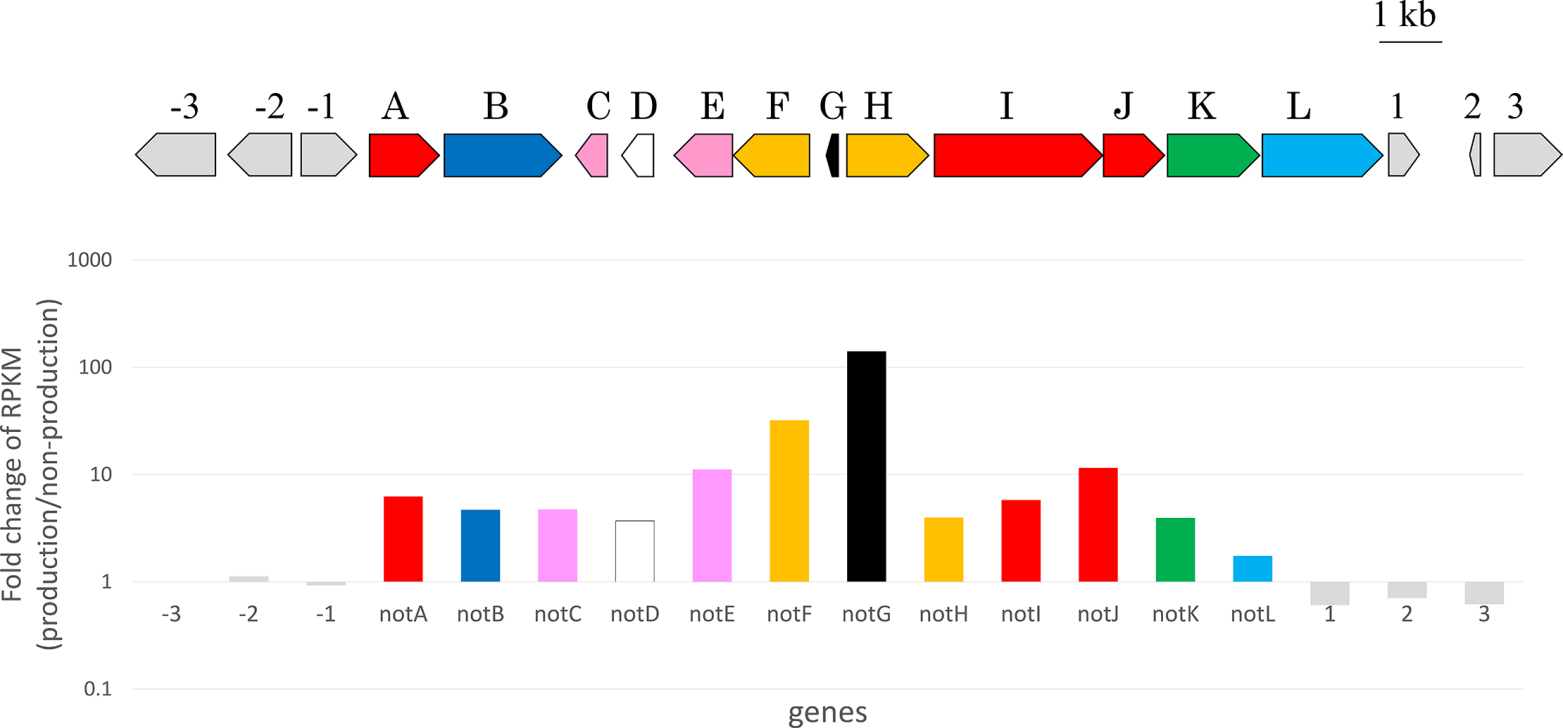
Transcriptional analysis. The transcription levels of genes in the nocardithiocin gene cluster were compared under nocardithiocin-producing and non-producing conditions. The RPKM values under nocardithiocin-producing conditions are relative to those under non-producing conditions. The graph of RPKM-fold change for each gene is shown under the diagram of the gene cluster.

Our preliminary study of cultivation conditions, revealed that addition of FBS to the RPMI 1640 medium improved nocardithiocin production in *N*. *pseudobrasiliensis* (S3 Fig). To further confirm the involvement of the predicted nocardithiocin biosynthetic gene cluster in nocardithiocin production, the expression levels of each genes was compared under nocardithiocin-producing (with FBS) and non-producing (without FBS) conditions. Expression levels were compared by reads per kilobase of exon per million mapped sequence read (RPKM) values by RNA-seq analysis. The RPKM is used to quantify gene expression from RNA-seq data by normalizing total read length and the number of mapped reads, so it can be used as the gene expression value. The fold changes in the RPKM values were calculated between nocardithiocin-producing and non-producing conditions (production/no-production). There were large RPKM-fold changes of the predicted genes within the predicted gene cluster (*notA*–*notL*), while the fold changes of the flanking genes were very small (Fig 5). The pattern of gene expression clearly changed in the predicted cluster. These results supported that the predicted 12 genes formed the cluster participate in nocardithiocin biosynthesis.

## Discussion

*Nocardia* species are Gram-positive soil bacteria and known opportunistic pathogens. Various species have been isolated from clinical samples [16–18]. Although *Nocardia* species are actinomycetes, there have been very few studies of their secondary metabolites, or the genes that synthesize them, in comparison with those conducted in *Streptomyces* species, mainly because of the lack of genetic tools. However, recent developments in *Nocardia*-*E*. *coli* shuttle vectors [19], and the availability of *Nocardia* genome sequences [3], prompted us to investigate the secondary metabolites of *Nocardia* species.

Nocardithiocin was purified from *N*. *pseudobrasiliensis* as a novel thiopeptide antibiotic compound [4]. Thiopeptides are ribosomally synthesized and post-translationally highly modified peptide compounds. Since the first thiopeptide compound was discovered, almost 100 additional related compounds have been identified. The wide range of bioactivities and efficient antimicrobial activities against drug-resistant pathogens, such as methicillin-resistant *S*. *aureus*, penicillin-resistant *S*. *pneumoniae*, and vancomycin-resistant enterococci, make thiopeptides attractive lead compounds for developing novel therapeutic drugs [5–9]. However, poor pharmacokinetics and low water solubility haveprevented thiopeptides from being used in humans [20]. To overcome these defects, chemical syntheses have been conducted to produce useful derivatives [21], and with the discovery of the thiopeptide biosynthetic gene cluster, gene replacement becomes a promising approach [6, 22].

In this study, we identified the nocardithiocin biosynthetic gene cluster in *N*. *pseudobrasiliensis* using the conserved sequence of an essential gene for thiopeptide biosynthesis (cyclodehydratase), along with whole genome sequence information. In the gene cluster, 12 genes, covering a 15.2-kb genomic region, were predicted to be involved in the nocardithiocin synthesis. Other reported thiopeptide gene clusters are in the range of 15.6−30.4 kb [10, 12–15], indicating our predicted cluster is a similar size. The genetic components of the predicted cluster were also similar to the previously reported thiopeptide gene clusters (S3A Fig). Previously reported precursor peptides are composed of 34−55 leading peptides at the N-terminus and 12−17 structural peptides at the C-terminus [5], and the representative structure of precursor peptides were shown in S3B Fig The precursor peptide of the predicted nocardithiocin gene cluster consisting of 46 leading peptides and 13 structural peptides, which agrees with these previous reports.

Gene disruption and complementation experiments demonstrated that the predicted gene cluster is responsible for nocardithiocin production. The disruption of a putative precursor peptide gene (*notG*) also resulted in abolished nocardithiocin production (data not shown). These results strongly suggest that the identified gene cluster is responsible for nocardithiocin production.

The cyclodehydratase gene fragment was successfully amplified from all 14 *N*. *pseudobrasiliensis* strains examined in this study, except IFM 0756, using degenerate primer set Thio-F and Thio-R. This supports the lack of nocardithiocin production observed in strain IFM 0756. Although strain IFM 10700 did not produce nocardithiocin, the cyclodehydratase gene was successfully amplified from this strain. DNA-sequencing analysis has not yet been conducted in these two nocardithiocin non-producing strains, so we cannot explain the reason for the lack of nocardithiocin production. However, a deletion of the cyclodehydratase gene or within the nocardithiocin gene cluster in IFM 0756, and a mutation in a critical gene other than cyclodehydratase, in IFM 10700 could be the cause of the lack of nocardithiocin production in these strain.

The recent advance in experimental techniques for transcriptomic analyses, including microarrays and RNA-seq, allowed us to quantitate gene expression levels. Generally, genes responsible for secondary metabolite production are adjacently located, and are coordinately regulated for appropriate metabolite production. The transcriptional analysis in this study clearly confirmed the initial and terminal genes within the cluster. Identifying the coordinating gene expression pattern by comparing the gene expression levels under different cultivation conditions, such as conditions for producing different secondary metabolites, could be a useful way to predict novel gene clusters for production of secondary metabolites in this microorganism.

Although nocardithiocin has a high level of antibiotic activity against rifampicin-resistant *M*. *tuberculosis*, the slight light sensitivity of this compound may prevent its clinical use [4]. The identification of the biosynthetic gene cluster expands the possibility for further structural modifications of nocardithiocin. Although further studies are needed, the genetic methodology used in this study along with the findings, might facilitate the use of *Nocardia* species as sources of secondary metabolites.

## Supporting Information

S1 Fig UV and MS spectra of nocardithiocin. (A) The UV spectrum of the nocardithiocin peak was obtained by HPLC detected with PDA. (B) Mass chromatogram of the position of the nocardithiocin HPLC peak. Mass spectra were obtained using a LTQ ORBITRAP XL mass spectrometer (Thermo Fisher Scientific, Kanagawa, Japan) equipped with HESI II positive-ion mode.
(PDF)

S2 Fig Comparison of the thiopeptide biosynthetic gene clusters. (A) Gene organization with the nocardithiocin and other reported thiopeptide biosynthetic gene clusters. (B) The amino acid sequence of precursor peptides of each thiopeptide.
(PDF)

S3 Fig Nocardithiocin production in RPMI 1640 medium with or without FBS. HPLC data for strains grown under nocardithiocin producing (FBS +) and non-producing (FBS -) conditions. The arrowhead indicates the peak corresponding to nocardithiocin.
(PDF)

## References

[1] Hwang KS, Kim HU, Charusanti P, Palsson BØ, Lee SY. Systems biology and biotechnology of *Streptomyces* species for the production of secondary metabolites. Biotechnol Adv. 2014;32(2): 255–268. 10.1016/j.biotechadv.2013.10.008 24189093

[2] Komatsu M, Uchiyama T, Omura S, Cane DE, Ikeda H. Genome-minimized *Streptomyces* host for the heterologous expression of secondary metabolism. Proc Natl Acad Sci U S A. 2010;107(6): 2646–2651. 10.1073/pnas.0914833107 20133795 PMC2823899

[3] Komaki H, Ichikawa N, Hosoyama A, Takahashi-Nakaguchi A, Matsuzawa T, Suzuki K et al Genome based analysis of type-I polyketide synthase and nonribosomal peptide synthetase gene clusters in seven strains of five representative *Nocardia* species. BMC Genomics. 2014;15: 323–333. 10.1186/1471-2164-15-323 24884595 PMC4035055

[4] Mukai A, Fukai T, Hoshino Y, Yazawa K, Harada K, Mikami Y. Nocardithiocin, a novel thiopeptide antibiotic, produced by pathogenic *Nocardia pseudobrasiliensis* IFM 0757. J Antibiot. 2009;62(11): 613–619. 10.1038/ja.2009.90 19745839

[5] Just-Baringo X, Albericio F, Álvarez M. Thiopeptide antibiotics: retrospective and recent advances. Mar Drugs.2014;12(1): 317–351. 10.3390/md12010317 24445304 PMC3917276

[6] Zhang F, Kelly WL. In vivo production of thiopeptide variants. Methods Enzymol. 2012;516: 3–24. 10.1016/B978-0-12-394291-3.00022-8 23034221

[7] Haste NM, Thienphrapa W, Tran DN, Loesgen S, Sun P, Nam SJ et al Activity of the thiopeptide antibiotic nosiheptide against contemporary strains of methicillin-resistant *Staphylococcus aureus* . J Antibiot. 2012;65(12): 593–598. 10.1038/ja.2012.77 23047246 PMC3528839

[8] Engelhardt K, Degnes KF, Kemmler M, Bredholt H, Fjaervik E, Klinkenberg G et al Production of a new thiopeptide antibiotic, TP-1161, by a marine *Nocardiopsis* species. Appl Environ Microbiol. 2010;76(15): 4969–4976. 10.1128/AEM.00741-10 20562278 PMC2916467

[9] Li W, Leet JE, Ax HA, Gustavson DR, Brown DM, Turner L et al Nocathiacins, new thiazolyl peptide antibiotics from *Nocardia* sp. I. Taxonomy, fermentation and biological activities. J Antibiot. 2003;56(3): 226–231. 12760678 10.7164/antibiotics.56.226

[10] Liao R, Duan L, Lei C, Pan H, Ding Y, Zhang Q et al Thiopeptide biosynthesis featuring ribosomally synthesized precursor peptides and conserved posttranslational modifications. Chem Biol. 2009;16(2): 141–147. 10.1016/j.chembiol.2009.01.007 19246004 PMC2676563

[11] Li C, Kelly WL. Recent advances in thiopeptide antibiotic biosynthesis. Nat Prod Rep. 2010;27(2): 153–164. 10.1039/b922434c 20111801

[12] Engelhardt K, Degnes KF, Zotchev SB. Isolation and characterization of the gene cluster for biosynthesis of the thiopeptide antibiotic TP-1161. Appl Environ Microbiol. 2010;76(21): 7093–7101. 10.1128/AEM.01442-10 20851988 PMC2976260

[13] Wang J, Yu Y, Tang K, Liu W, He X, Huang X et al Identification and analysis of the biosynthetic gene cluster encoding the thiopeptide antibiotic cyclothiazomycin in *Streptomyces hygroscopicus* 10–22. Appl Environ Microbiol. 2010;76(7): 2335–2344. 10.1128/AEM.01790-09 20154110 PMC2849233

[14] Ding Y, Yu Y, Pan H, Guo H, Li Y. Moving posttranslational modifications forward to biosynthesize the glycosylated thiopeptide nocathiacin I in *Nocardia* sp. ATCC202099. Mol Biosyst. 2010;6(7): 1180–1185. 10.1039/c005121g 20473441

[15] Morris RP, Leeds JA, Naegeli HU, Oberer L, Memmert K. Ribosomally synthesized thiopeptide antibiotics targeting elongation factor Tu. J Am Chem Soc. 2009;131(16): 5946–5955. 10.1021/ja900488a 19338336

[16] Muñoz J, Mirelis B, Aragón LM, Gutiérrez N, Sánchez F, Español M et al Clinical and microbiological features of nocardiosis 1997–2003. J Med Microbiol. 2007;56: 545–550. 17374898 10.1099/jmm.0.46774-0

[17] Minero MV, Marín M, Cercenado E, Rabadán PM, Bouza E. Nocardiosis at the turn of the century. See comment in PubMed Commons belowMedicine (Baltimore). 2009;88(4): 250–261. 10.1097/MD.0b013e3181afa1c8 19593231

[18] Wilson JW. Nocardiosis: Updates and Clinical Overview. Mayo Clin Proc. 2012;87(4): 403–407. 10.1016/j.mayocp.2011.11.016 22469352 PMC3498414

[19] Chiba K, Hoshino Y, Ishino K, Kogure T, Mikami Y. Construction of a pair of practical *Nocardia*-*Escherichia coli* shuttle vectors. Jpn J Infect Dis. 2007;60(1): 45–47. 17314425

[20] Bhansali SG, Mullane K, Ting LS, Leeds JA, Dabovic K. Pharmacokinetics of LFF571 and vancomycin in patients with moderate *Clostridium difficile* infections. Antimicrob Agents Chemother. 2015;59(3):1441–1445. 10.1128/AAC.04252-14 25534724 PMC4325791

[21] Xu L ^1^, Farthing AK, Dropinski JF, Meinke PT, McCallum C, Hickey E et al Synthesis and antibacterial activity of novel water-soluble nocathiacin analogs. Bioorg Med Chem Lett. 2013;23(1): 366–369. 10.1016/j.bmcl.2012.10.065 23164707

[22] Bowers AA, Acker MG, Koglin A, Walsh CT. Manipulation of thiocillin variants by prepeptide gene replacement: structure, conformation, and activity of heterocycle substitution mutants. J Am Chem Soc. 2010;132(21): 7519–7527. 10.1021/ja102339q 20455532 PMC2882596

