# Supplementary figures and images for: Identification and Functional Analysis of the Nocardithiocin Gene Cluster in Nocardia pseudobrasiliensis
Source: PLoS One. 2015 Nov 20;10(11):e0143264. doi: 10.1371/journal.pone.0143264 (PMC4654471; doi:10.1371/journal.pone.0143264)

S1 Fig.     UV and MS spectra of nocardithiocin

A

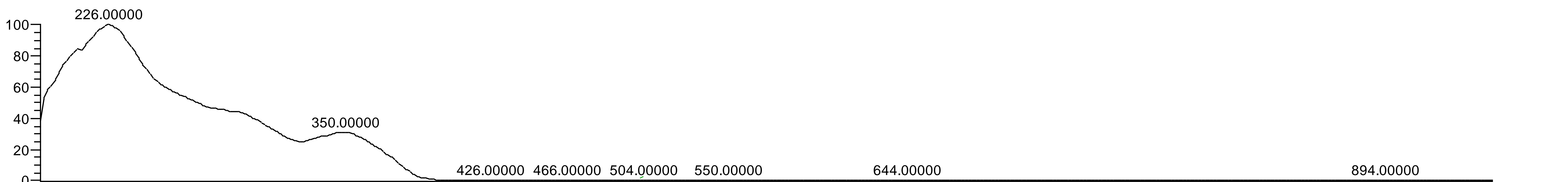

B

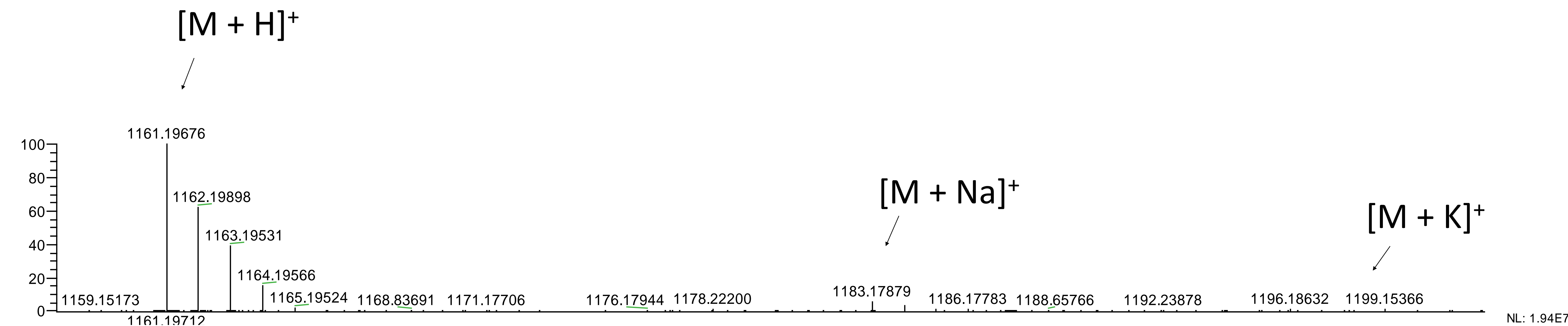

|                     |                      |
|---------------------|----------------------|
| Calculated MS (m/z) | 1161.19625 $[M+H]^+$ |
| Measured MS (m/z)   | 1161.19676 $[M+H]^+$ |

Supplement: S1 Fig — (A) The UV spectrum of the nocardithiocin peak was obtained by HPLC detected with PDA. (B) Mass chromatogram of the position of the nocardithiocin HPLC peak. Mass spectra were obtained using a LTQ ORBITRAP XL mass spectrometer (Thermo Fisher Scientific, Kanagawa, Japan) equipped with HESI II positive-ion mode. (PDF) [file pone.0143264.s001.pdf]

S3 Fig. Nocardithiocin production in RPMI 1640 medium with or without FBS

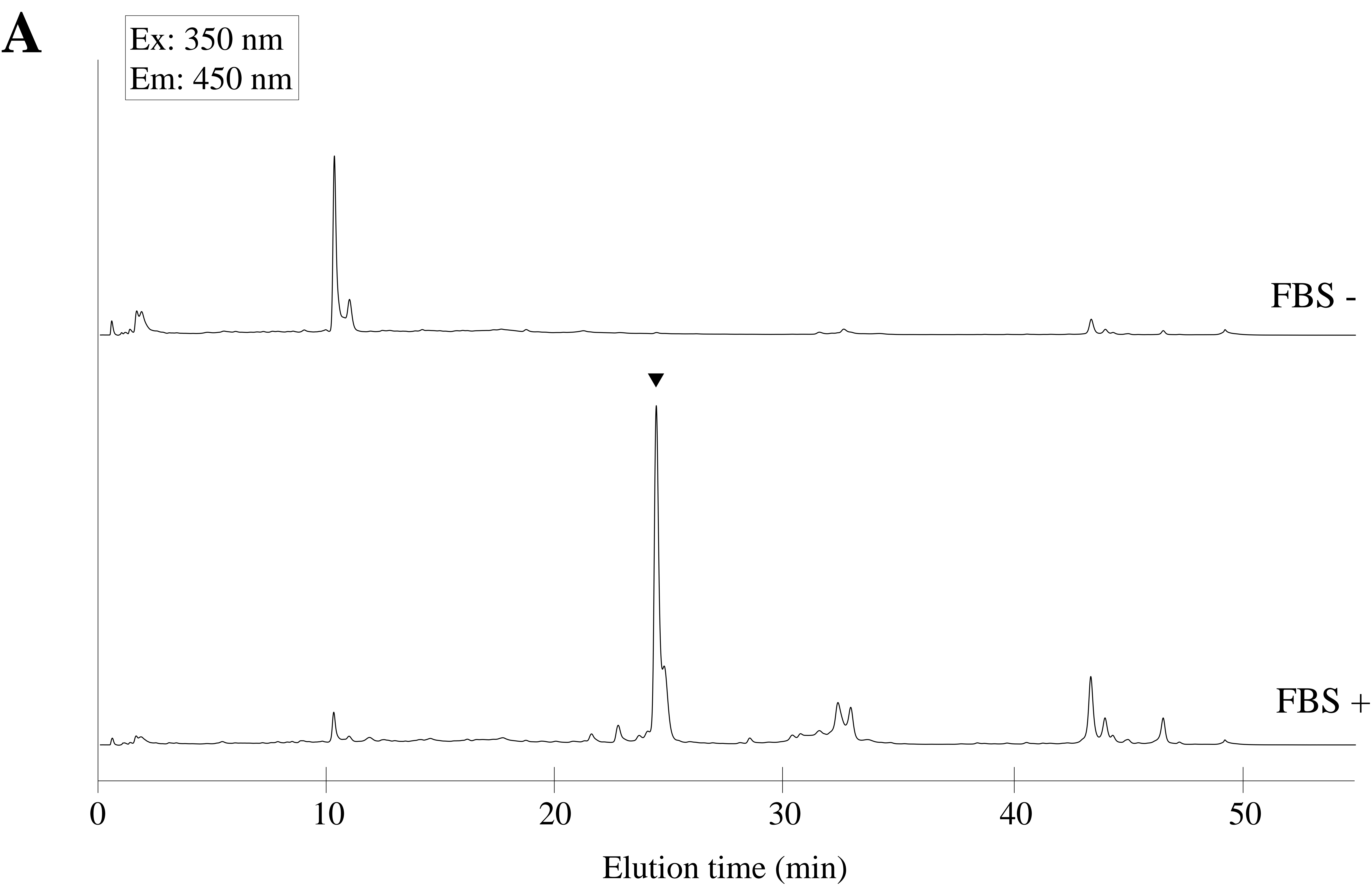

Supplement: S3 Fig — HPLC data for strains grown under nocardithiocin producing (FBS +) and non-producing (FBS -) conditions. The arrowhead indicates the peak corresponding to nocardithiocin. (PDF) [file pone.0143264.s003.pdf]
