# Supplementary material for: Identification and Functional Analysis of the Nocardithiocin Gene Cluster in Nocardia pseudobrasiliensis
Source: PLoS One. 2015 Nov 20;10(11):e0143264. doi: 10.1371/journal.pone.0143264 (PMC4654471; doi:10.1371/journal.pone.0143264)
Supplement: S2 Fig — (A) Gene organization with the nocardithiocin and other reported thiopeptide biosynthetic gene clusters. (B) The amino acid sequence of precursor peptides of each thiopeptide. (PDF) [file pone.0143264.s002.pdf]

S2 Fig.

Comparison of the thiopeptide biosynthetic gene clusters

A

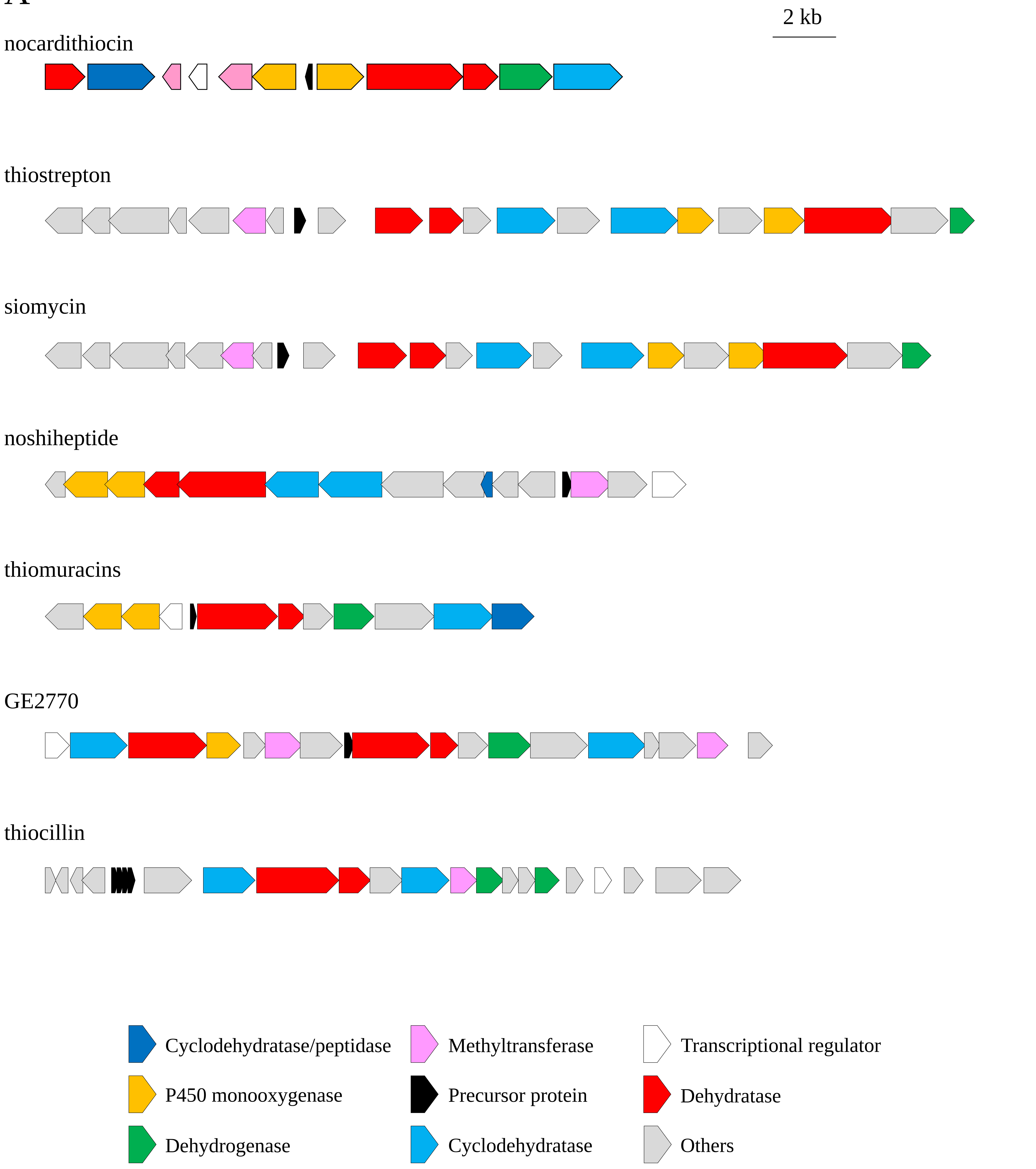

S2 Fig.

Comparison of the thiopeptide biosynthetic gene clusters

B

	Leader peptide	Precursor peptide
NotG (nocardithiocin)	MSAQDKDPNEIRRRFEELPMEVFQLDGSGLPIESLTDGHGMTEVGA	**SCTSCVCICSCCT**
TsrA (thiostrepton)	MSNAALEIGVEGLTGLDVDTLEISDYMDETLLDGEDLTVTM	**IASASCTTCICTCSCSS**
SioH (siomycin)	MSTAAIVGQEIGVDGLTGLDVDALEISDYMDETLLDGEDLSVTM	**VSSASCTTCICTCSCSS**
NosM (nosiheptide)	MDAAHLSDLDIDALEISEFLDESRLEDSEVVAKVMSA	**SCTTCECCCSCSS**
TpdA (thomuracins)	MDLSDLPMDVFELADDGVAVESLTAGHGMTEVGA	**SCNCFCYICCSCSSA**
TpdA (GE2270)	MSELESKLNLSDLPMDVFEMADSGMEVESLTAGHGMPEVGA	**SCNCVCGFCCSCSPSA**
TclE-H (thiocillin)	MSEIKKALNTLEIEDFDAIEMVDVDAMPENEALEIMGA	**SCTTCVCTCSCCTT**
